# Effect of universal adhesives on microtensile bond strength to hybrid ceramic

**DOI:** 10.1186/s12903-019-0865-7

**Published:** 2019-08-06

**Authors:** Mohamed M. Awad, Lamees Albedaiwi, Ahmed Almahdy, Rawaiz Khan, Nick Silikas, Muhanad M. Hatamleh, Fahad M. Alkhtani, Ali Alrahlah

**Affiliations:** 1 Department of Conservative Dental Sciences, College of Dentistry, Prince Sattam Bin Abdulaziz University, Alkharj, 11942 Saudi Arabia; 2 Preventive Dental Program, Ministry of Health, Riyadh, 11179 Saudi Arabia; 3 Department of Pediatric Dentistry and Orthodontics, College of Dentistry, King Saud University, Riyadh, 11545 Saudi Arabia; 4 Engineer Abdullah Bugshan research chair for Dental and Oral Rehabilitation, King Saud University, Riyadh, 11545 Saudi Arabia; 5 Dentistry, School of Medical Sciences, University of Manchester, Manchester, UK; 6 Luminous Technical University College, Amman, Jordan; 7 Department of Prosthodontics, College of Dentistry, Prince Sattam Bin Abdulaziz University, Alkharj, 11942 Saudi Arabia; 8 Department Of Restorative Dental Sciences, College of Dentistry, King Saud University, Riyadh, 11545 Saudi Arabia

**Keywords:** Universal adhesive, Ceramic, Bond strength, Silanization

## Abstract

**Background:**

The aim of this study was to evaluate the effect of universal adhesives (UA) and silane on the microtensile bond strength (μTBS) of resin cement to a hybrid ceramic Vita Enamic (VE).

**Methods:**

VE specimens were acid etched using hydrofluoric acid (HF) and were assigned to three groups (*n* = 10) based on the applied bonding technique. In group 1 (S), a silane-based primer was used as a surface treatment prior to the application of a resin cement (Variolink Esthetic DC). In group 2, a silane-containing UA, Clearfil Universal Bond (CUB) was used for the surface treatment, and in group 3, A silane-free UA, Tetric N-Bond Universal (TNU) was used for surface treatment. Resin cement build-ups were prepared. The bonded specimens were sectioned into resin-ceramic beams. Half of the beams of each group were stored for 24 h at 37 °C and the other half were subjected to a thermo-cycling aging. The microtensile bond strength (μTBS) was measured at a crosshead speed of 0.5 mm/min. Failure modes were assessed accordingly. Data were analyzed using a) two-way analysis of variance ANOVA followed by one-way ANOVA and Tukey tests between groups and b) independent *t-*test to detect differences (α = 0.05) for each group. The surface topographies of the ceramic surface were evaluated using scanning electron microscopy.

**Results:**

The results showed that silane-based primer (S) application resulted in significantly higher *(p < 0.05)* μTBS values after 24 h and after thermocycling compared to both silane-containing UA (CUB) and silane-free UA (TNU). The μTBS values of all groups were significantly reduced after thermocycling. No statistically significant difference was observed between the μTBS of CUB and TNU after 24 h. However, TNU showed significantly higher μTBS after thermocycling. Different failure modes were observed, and adhesive failure was the most common in all groups. Marked surface topographic changes were observed following HF etching.

**Conclusion:**

It is concluded that, the UAs tested cannot be recommended as substitutes to the silanization of Hybrid ceramic.

## Background

Dental ceramic materials are widely applied in indirect esthetic restorations using computer-aided design and computer-aided manufacturing (CAD/CAM) technology [1, 2]. From the material prescriptive, dental ceramics have recently been classified into three main categories: glass-ceramics; polycrystalline ceramics; and resin-matrix ceramics, also known as hybrid ceramics (HCs) [3]. Vita Enamic (VE), Vita Zahnfabrik; Bad Säckingen, Germany, is one example of HCs. VE consists of inorganic phase and organic phase, in which a dominant ceramic network is reinforced by an acrylic polymer network resin, with both networks fully penetrating into one another [4]. HC restorations have shown promising performances in in-vitro [5–9] and clinical studies [10].

Reliable bonding is essential for the long-term clinical success of ceramic restorations [11, 12]. This bonding can be affected by the surface treatment of the ceramic substrate [13], and the chemistry of the selected adhesive [14]. Surface treatments of dental ceramics depend on their compositions [15]. For glass-ceramics restorations, it is recommended to etch the intaglio surface using hydrofluoric acid (HF) to create surface micro irregularities [16, 17], before the application of the 3-methacryloxypropyltrimethoxysilane (silane)-based primer which can enhance the physical and chemical bonding between the glass-ceramics and methacrylate-based resin materials such as resin cements [18]. The adhesive strategy of glass-ceramics is applied to HCs.

Universal adhesives (UAs) are the latest category of dental adhesives [19]. The majority of commercial UAs contain 10-methacryloyloxydecyl dihydrogen phosphate (MDP), however a few contain both MDP and silane. UAs are claimed to promote bonding to dental substrates including various ceramics. UAs enhanced the bonding to polycrystalline ceramics such as zirconia because of the presence of MDP [20–22], and showed promising results towards the improvement the bonding strength to indirect resin-based composite [23]. By contrast, with regard to glass-ceramics such as Lithium disilicate ceramic, the application of UAs has failed to replace the silane-based primer [23, 24]. Currently, there is a lack of information regarding the effectiveness of UAs as substitutes to the silane-based primer, specifically with respect to the bonding of HCs. Therefore, the objective of this study was to investigate the effect of UAs, as substitutes to the silane-based primer application, on microtensile bond strength (μTBS) of the resin cement to the VE, and to evaluate the surface topography of VE after HF etching. The null hypotheses were as follow 1) there is no difference in the μTBS of the resin cement to the VE following the application of silane-based primer or UAs at 24 h and after thermocycling, and 2) there is no difference in μTBS following the application of silane-containing or silane-free UAs at 24 h and after thermocycling.

## Methods

The materials used in this study and their specifications are summarized in Table 1. Thirty Vita Enamic©, Vita Zahnfabrik, Bad Säckingen, Germany (VE) blocks (with dimensions of 6 × 6 × 6 mm^3^) were made using a low-speed cutting machine (Isomet, *Buehler Ltd., USA*), with a 4-in. circular diamond wheel (MetLab Technologies Limited, UK) and water coolant. The blocks were cleaned ultrasonically in distilled water for 5 min and the top surface of each block was standardized using #600 silicon carbide papers (CrbiMet Abrasive Disks, Buehler, Lake Bluff, IL, USA) on a 300 rev/min grinding machine (Automata, Jean Wirtz, Dusseldorf, Germany) for 1 min, followed by a) ultrasonic cleaning in distilled water for 5 min using an ultrasonicator (Sonicer, Yoshida Dental Manufacturing. Co., Ltd., Tokyo, Japan), and b) air-dring.

**Table 1:** Materials composition and supplier’s details

Material	Brand name, manufacturer and LOT number	Composition	Instructions of use
Hybrid ceramic	Vita Enamic (VE) (Vita Zahnfabrik; Bad Säckingen, Germany) LOT41110	SiO_2,_ Al_2_O_3,_ Na_2_O, K_2_O, B_2_O_3,_ ZrO_2,_ CaO, UDMA, TEGDMA	
Silane-based primer	Silane (S) (Silane, Pulpdent Corporation, Watertown, MA, USA) 02471	MPS, 2-Propanol	1. Apply silane using micro-brush 2. Let evaporate for 1 min 3. Blow with gentle air stream until completely dry
Ceramic etchant	IPS Ceramic (Ivoclar Vivadent, Schaan, Liechtenstein) U39349	4.6% Hydrofluoric acid	
Universal adhesives	Clearfil Universal Bond (CUB) (Kuraray Noritake Dental Inc. Chiyoda Ku, Tokyo, Japan) 000001	Bis-GMA, HEMA, ethanol, 10-MDP, hydrophilic aliphatic dimethacrylate, colloidal silica, DL camphorquinone, silane coupling agent, accelerators, initiators, water	1. Dispense one drop each of BOND and “CLEARFIL DC Activator” into a well of the dispensing dish and mix them together with the applicator brush. 2. Apply the mixture then brush and leave it for 5 s. 3. Dry by blowing mild air for more than 5 s until the adhesive shows no move.
	Tetric N-Bond Universal (TNU) (Ivoclar Vivadent, Schaan, Liechtenstein) U22452	MDP, MCAP, HEMA, D3MA water, ethanol, highly dispersed silicon dioxide, initiators and stabilizers	1. Apply and then scrub for at least 20 s 2. Disperse with oil- and moisture-free compressed air until a glossy, immobile film layer.
Resin cement (Dual cure)	Variolink Esthetic DC Vivadent, Schaan, Liechtenstein) V19788	urethane dimethacrylate, methacrylate monomers, inorganic fillers (ytterbium trifluoride and spheroid mixed oxide), Initiators, stabilizers and pigments	1. Dispense Variolink Esthetic DC from the automix syringe 2. Apply Variolink Esthetic DC directly to surface treated ceramic material. 3. Light cure

### Bonding procedure

The HF (4.6%) acid was applied to the top surface of the prepared VE blocks using a plastic application tip for 60s to create an etched surface. Etched surfaces were then washed using water for 30s and then air-dried until no moisture was visible. The VE blocks were randomly allocated into three experimental groups (*n* = 10) based on the bonding technique used. In group 1 (S), a silane-based primer (Silane, *Pulpdent Corporation, Watertown, MA, USA*) was applied to the etched VE using a micro-brush, let evaporate for 1 min, and blown with gentle air stream until it was completely dried. In group 2, a silane-containing UA, Clearfil Universal Bond (CUB) (*Kuraray Noritake Dental Inc., Japan*), was mixed with Clearfil DC Activator (*Kuraray Noritake Dental Inc., Japan*) according to manufacturer instructions, and used for surface treatment. In group 3; A silane-free UA, Tetric N-Bond Universal (TNU), (*Ivoclar Vivadent, Schaan, Liechtenstein*), was used as a surface treatment. Both UAs were rubbed on the VE surface for 20s, and were air-dried until a glossy, immobile film layer is formed. However, light-curing of UAs was avoided.

Following the use of Silane and UAs, dual-cure resin luting cement (Variolink esthetic DC, *Ivoclar Vivadent, Schaan, Liechtenstein*) build-ups were prepared. Resin cement was applied incrementally and was adapted using a silicon mold (6 × 6 × 12 mm), which matched the dimensions of VE blocks, thus, a 6 × 6 × 6 mm^3^ resin cement build-up was allowed on the top surface on each of the VE blocks. Each resin cement increment was light cured for 40s using a light emitting diode light curing unit (Bluephase®, Ivoclar Vivadent, Austria) operated at a light irradiance of 1000 mW/cm^2^ as measured using a digital radiometer (Bluephase Meter, Ivoclar Vivadent, Austria). Moreover, to ensure adequate polymerization, additional light curing was performed at four different directions was carried out for 40s after the mold was removed. The tip of light-curing unit was kept as close as possible (approximately 1 mm), and at zero angle without touching the specimens. This bonding procedure was implemented in all the groups by one operator. Specimens were stored in distilled water for 24 h at 37 °C.

### μTBS test

The bonded blocks were vertically sectioned into serial slabs. These were then sectioned into beams with a cross-sectional area of 1 mm^2^ (±0.1), using a low-speed cutting saw with a water-cooled diamond blade. Peripheral beams were not used in this study as the margins of the VE blocks may have been ineffectively acid etched with HF. Specimens from each group were subdivided into two sub-groups. Half of the specimens were tested immediately after 24 h of storage in distilled water, while the other half was tested after 5000 cycles of thermocycling using thermocycler machine (THE-1100, SD Mechatronik GmbH, Germany). In each cycle, the specimens were placed in a water bath at 55 °C for 30 s. Subsequently, they were placed in a water bath at 5 °C for 30s.

The cross-sectional area of each bonded stick was measured using a digital caliber. The microtensile bond strength (μTBS) test was carried out using a universal testing machine (Instron 5965, Instron Corporation, USA) with a load cell of 5kN and a cross-speed of 0.5 mm/min. Tensile stress was applied to bonded beams until failure. The μTBS (expressed in MPa) was measured by dividing force in Newtons by premeasured surface area in mm^2^.

After the debonding, fractured beams were vertically mounted onto coded brass stubs, and the failure modes were assessed using a scanning electron microscopy (SEM) (JSM-6360LV, JEOL Ltd., Tokyo, Japan) at × 50 magnification operated at 15 kV. The failure modes were classified as follows: adhesive failure, cohesive failure in the ceramic, cohesive failure in the resin-cement, and mixed failure (adhesive failure together with cohesive failure in the cement).

### Evaluation of surface topography

To evaluate the surface morphology of VE, four discs (6 × 6 mm) were prepared by cutting the VE blocks. The top surfaces of the VE blocks were standardized and ultrasonically cleaned using the previously mentioned protocol, and then set into two groups (*n* = 2); in the first group (unetched) and the second group (HF-etched); in which VE was acid-etched using HF acid as previously described. Specimens of both groups were gold-coated using a sputter coater (fine coat ion sputter JFC-1100, JEOL Ltd., Tokyo, Japan) for 180 s at 40 mA. The specimens were then mounted onto coded brass stubs and examined using SEM (JSM-6360LV, JEOL Ltd., Tokyo, Japan), at × 1500 magnification) operated at 15 kV.

### Statistical analyses

The statistical unit of analysis was the block (average of the beams tested). Data were analyzed using two-way analysis of variance ANOVA followed by one-way ANOVA and Tukey’s *post-hoc* tests between groups and with independent *t*-tests to detect differences (α = 0.05) for each group.

## Results

**Fig. 1:**
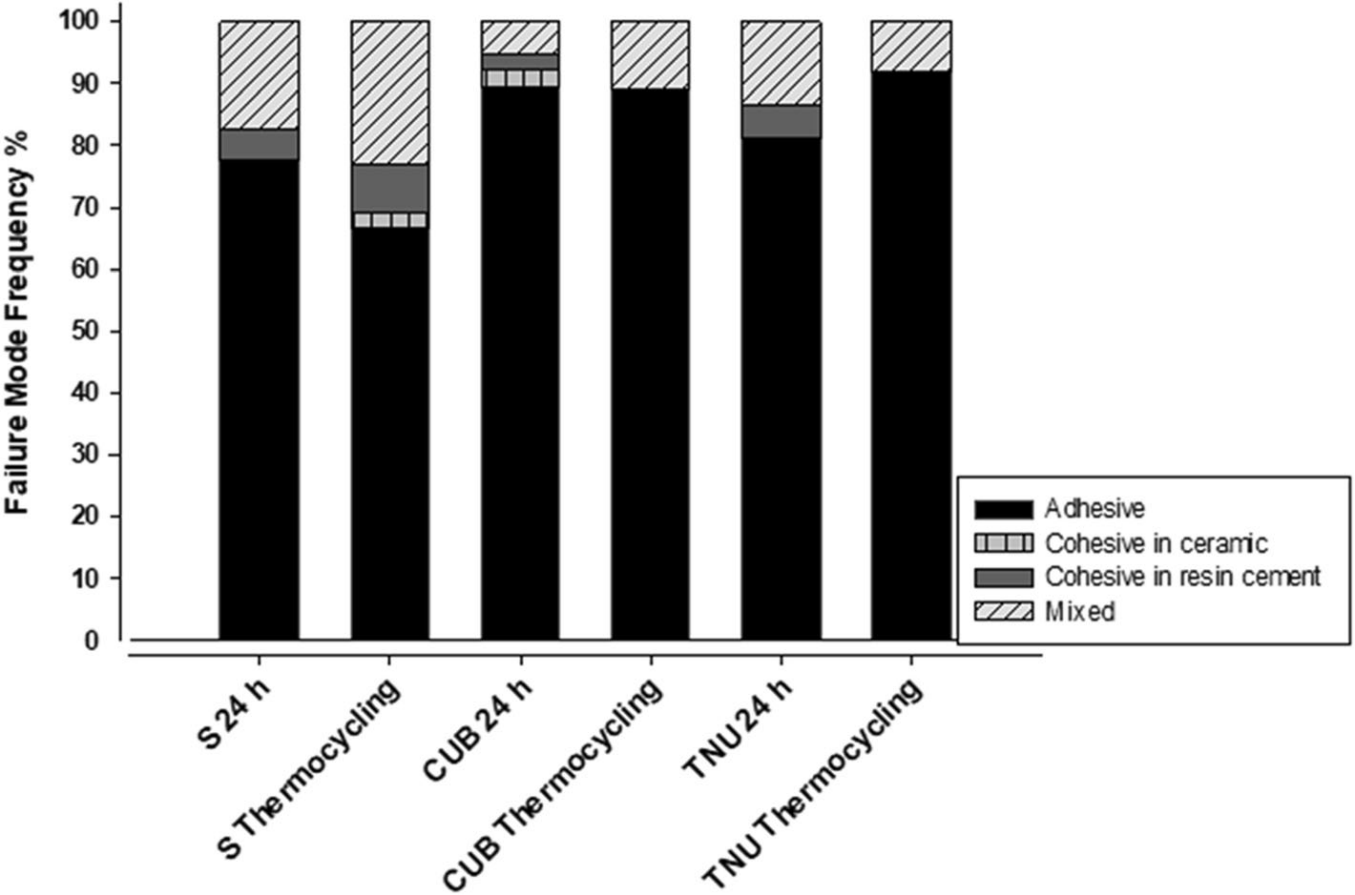
Frequency (%) of failure modes observed

The means and standard deviations of the μTBS, in MPa, are shown in Table 2. The numbers of beams tested for each group at 24 h and after thermocycling were (S:45, CUB:44, TNU:43) and (S:44, CUB:43, TNU:43) respectively. Silane-based primer (S) application resulted in significantly higher μTBS values (*p* < 0.05) after 24 h (59.14 ± 3.19 MPa) and after thermocycling (33.18 ± 2.37 MPa) as compared to silane-containing UA (CUB: 29.66 ± 1.07; 15.76 ± 1.95 MPa) and silane-free UA (TNU: 29.65 ± 1.21; 17.81 ± 1.19 MPa). Even though the μTBS values of all groups were significantly reduced after thermocycling, there was no statistically significant difference between the bond strength of CUB and TNU after 24 h (*p* > 0.05). However, TNU yielded significantly higher μTBS values compared to CUB after thermocycling (*p* < 0.05). There were statistically significant interactions between the surface treatments and storage times (*p* < 0.001). The frequencies of the failure modes observed are presented in Fig. 1. Adhesive, mixed, and cohesive failure modes were categorized with adhesive interface failure as the most common failure mode detected in all the tested groups at 24 h; (S: 77.5%, CUB: 89.5%, TNU:81.1%) and after thermocycling (S:66.7%, CUB:89.2%, TNU:91.9%).

**Table 2:** Mean (Standard Deviation) of μTBS values (MPa) of tested groups. The different superscript small letters indicate significant differences between groups in a same column *p* < 0.05 * indicated significant differences between storage time for the same group (rows) *p* < 0.05

Group	μTBS after 24 h	μTBS after thermocycling
S	59.14 ^a^ (3.19)	33.18 ^a^ (2.37) ^*^
CUB	29.66 ^b^ (1.07)	15.76 ^b^ (1.95) ^*^
TNU	29.65 ^b^ (1.21)	17.81 ^c^ (1.19) ^*^

**Fig. 2:**
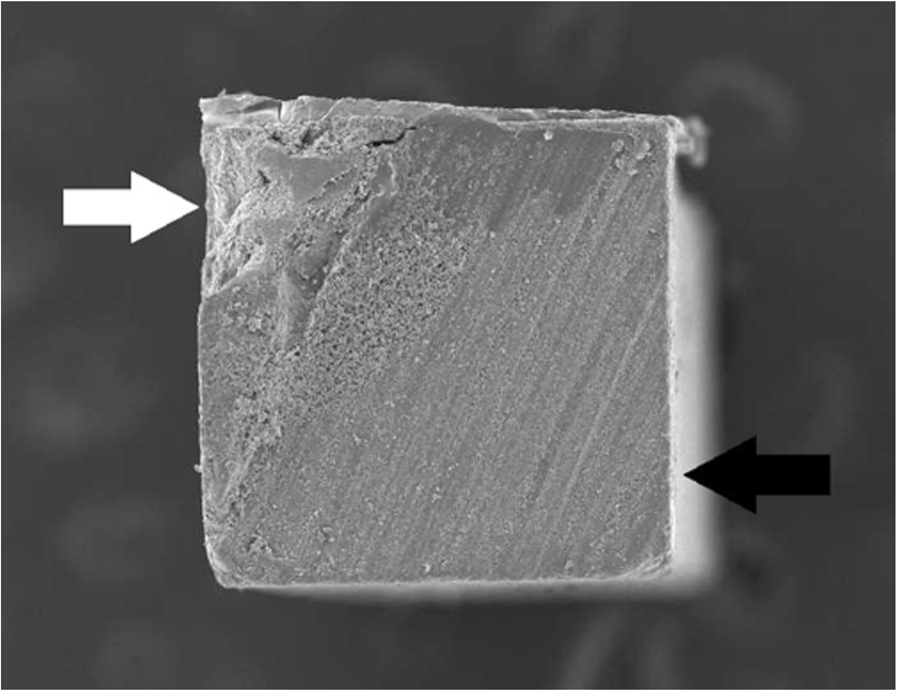
SEM micrograph (× 50 magnification) of mixed failure showing resin cement (white arrow) and VE (black arrow)

Mixed failure (Fig. 2) was the second most common failure pattern in all the groups at 24 h; (S: 17.5%, CUB: 5.3%, TNU:13.5%) and after thermocycling (S:23.1%, CUB: 10.8%, TNU:8.1%). Cohesive failure in resin cement was detected in all groups at 24 h; (S:5%, CUB: 2.6%, TNU: 5.4%) and after thermocycling (S:7.6%, CUB:0%, TNU:0%). Cohesive failure in ceramic was the least frequently detected failure pattern (2.6%) and was only observed in CUB group at 24 h and (S) after thermocycling. No pretest failures were detected in all groups.

**Fig. 3:**
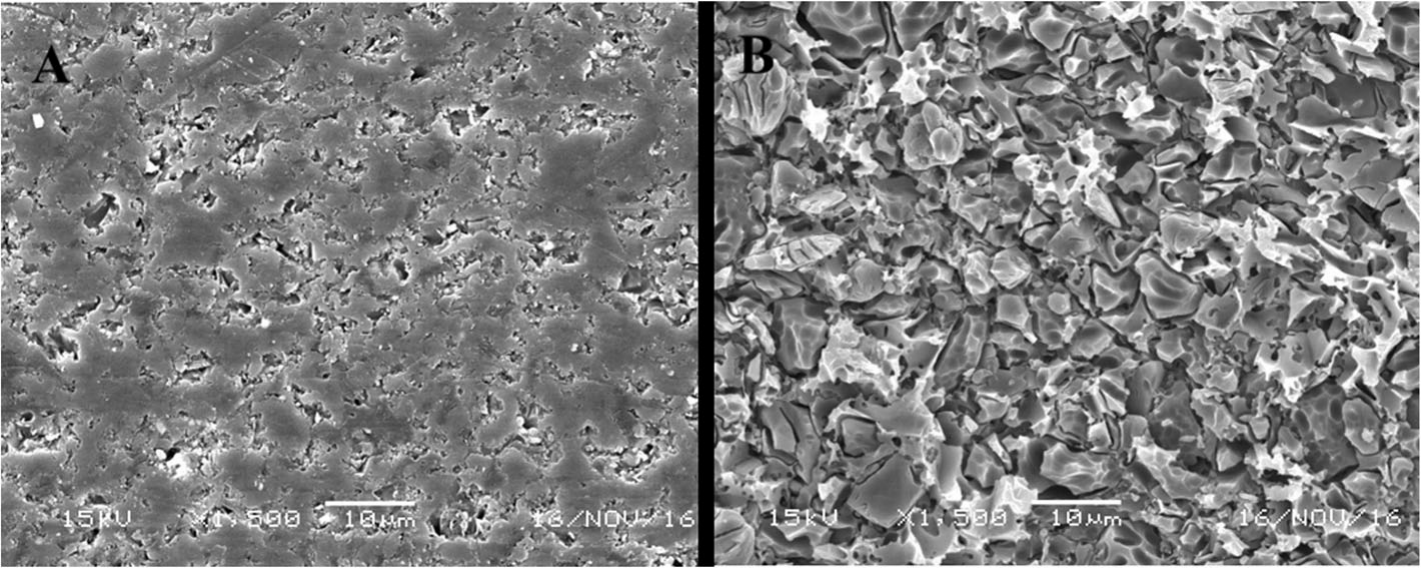
SEM micrograph (× 1500 magnification) of **a** unetched and **b** HF-etched VE

SEM micrographs (× 1500 magnification) showed that, the VE samples etched with HF exhibited marked change in surface morphology, higher retentive shadow irregularities and more prominent crystals (Fig. 3b) in comparison to the unetched samples. These revealed smooth and homogenous surface with fewer crystals that were less prominent, and with very less retentive irregularities (Fig. 3a).

## Discussion

The present study investigated the effect of silane-containing and silane-free UAs on the bonding of resin cement to VE. All groups were subjected to HF (4.6%) etching. HF can be used to dissolve the ceramic glass phase surface via its reaction with silicon dioxide [25]. HF etching results in enlarged surface texture and increased surface micro-irregularities [11, 25–27] increases the surface energy of the ceramic and reduces the contact angle for bonding agents. [28] SEM examinations (Fig. 3b) showed marked changes in surface morphology, increased surface micro-irregularities and randomly distributed micropores after HF etching. Similar features were observed in previous studies [29, 30]. The silane-based primer was used in group S as per manufacturer’s recommendations. Silane increases the chemical adhesion between the ceramic and resin materials [31, 32]. Silane molecules react with water to form three silanol groups from the corresponding methoxy groups [31]. The silanol groups thereafter react to form a siloxane network with the silica surface. The monomeric ends of the silane molecules react with the methacrylate groups of the resins via a free radical polymerization process [31]. Silane-containing and silane-free UAs were applied in groups CUB and TNU respectively.

The μTBS results showed that the application of the silane-based primer resulted in significantly higher mean bond strength compared to the silane-containing UA and silane-free UA at 24 h and after thermocycling. Therefore, the first hypothesis was rejected. These results may be explained by the lack of chemical adhesion similar to promoted by silane, between the UAs and VE. The pH of CUB is acidic (2.3) and that may render silane unstable [33]. and less effective for the formation of a strong siloxane network [34]. Moreover, the complex adhesive composition may negatively affect the reaction of silane to the glass-ceramics [35]. Additionally, the high viscosity of the adhesive solution -compared to the silane-based primer- may reduce the penetrative effects of the adhesive on the surface irregularities of the etched ceramic [35]. However, additional studies may be required to confirm this explanation. The MDP content of both UAs, as well as resin content in VE, did not appear to influence the bonding between UAs and HC. Accordingly, the MDP may not contribute to bonding to the glass- ceramics [34], and the resin content of VE exhibits a higher degree conversion and lacks unreacted monomers. There was no statistically significant difference between the bond strength of CUB and TNU after 24 h. Hence, the second hypothesis was partially rejected.

Different failure modes were observed in this study, with adhesive failure being the most common in all groups. This may be explained by the uniform and homogeneous stress distribution during μTBS testing, and the small surface areas of the bonded interfaces (approximately 1 mm^2^) of the tested beams [36]. The lack of pretest failures in the three experimental conditions may be explained by the adequate μTBS values obtained herein. In part, this relied on the surface micro-irregularities and topographic changes created (Fig. 3b) following HF etching.

Thermo-cycling procedures for age testing influence the bond strength of bonded materials [37]. The μTBS values of all the tested groups were significantly reduced after thermocycling. Water storage and thermocycling both result in the hydrolytic degradation of the ceramic/resin interface [38]. Thermo-cycling also typically results in combined contraction/expansion stresses and accelerated chemical degradation [39].

Based on the composition similarities, μTBS at 24 h, and the failure modes observed for the two methacrylate-based UAs, it can be assumed that, the use of the same manufacturer UA and resin cement may have a minimal effect on μTBS. However, TNU was more resistant compared to CUB, that may be attributed to hydrophobic crosslinking dimethacrylate decandiol dimethacrylate (D3MA). Hydrophobic monomers may be more resistant to degradation and should make the interface more hydrophobic with direct benefit to the bond stability [40].

The results of the present study are in agreement with the findings of previous studies [24], and suggest that the use of a silane-containing UA is not suitable as a substitute for the separate application of silane-based primer. The results of this study are also in accordance with the findings of previous in vitro studies [29, 30, 41, 42] and with recent guidelines published by The International Academy for Adhesive Dentistry [43]. These guidelines considered the use of HF acid etching followed by the addition of silane, to be the bonding strategy of choice for HC materials.

## Conclusion

Within the limitations of this study, the Universal Adhesives tested cannot be recommended as substitutes to the silanization of hybrid ceramic material.

## Data Availability

All the data generated or analyzed during this study are included in this published article.

## Abbreviations

CUB: Clearfil Universal Bond
HF: Hydrofluoric acid
MDP: 10-methacryloyloxydecyl dihydrogen phosphate
SEM: Scanning electron microscope
TNU: Tetric N-Bond Universal
UA: Universal adhesives
VE: Vita Enamic
μTBS: Microtensile bond strength
